# Effect of Pesticides on Biological Control Potential of *Neoscona theisi* (Araneae: Araneidae)

**DOI:** 10.1093/jisesa/iez024

**Published:** 2019-03-27

**Authors:** Hafiz Muhammad Tahir, Tayyba Basheer, Shaukat Ali, Rabia Yaqoob, Sajida Naseem, Shafaat Yar Khan

**Affiliations:** 1 Department of Zoology, GC University Lahore, Lahore, Pakistan; 2 Department of Zoology, University of Education, DG Khan Campus, Dera Ghazi Khan, Pakistan; 3 Department of Zoology, University of Education, Lower Mall Campus, Lahore, Pakistan; 4 Department of Zoology, University of Sargodha, Sargodha, Pakistan

**Keywords:** pesticide, spider, biological control, toxicity, herbicide

## Abstract

The present study was designed to record the effect of λ-cyhalothrin, Bifenthrin, and Glyphosate on the mortality, avoidance behavior, foraging activity, and activity of Acetylcholine esterase (AChE) and Carboxylesterase (CarE) in *Neoscona theisi* (Walckenaer, 1841). Highest mortality (70%) in *N. theisi* was recorded against λ-cyhalothrin. However, Glyphosate was found to be least toxic. Spider spent less time on insecticides/herbicide-treated surfaces. Insecticides/herbicide-treated *N. theisi* consumed less prey than untreated control spiders. Similarly, when *N. theisi* were offered insecticide/herbicide-treated prey, they consumed significantly less. Increased AChE and CarE activities were recorded in insecticides/herbicide-treated spiders as compared to control group. Total protein contents were less in insecticides/herbicide-treated spiders than control group. The results revealed that λ-cyhalothrin is more harmful to spiders as compared to Bifenthrin and Glyphosate. It is suggested that the effect of all pesticides used in agro-ecosystem on beneficial insects should be evaluated before using them in the fields.

Spiders (Arachnida: Araneae) are the most important group of natural predators in the agro-ecosystem representing about 47,771 described species (WSC 2018). They are highly diversified and significantly suppress insect pests in different agricultural fields (Anis Joseph and Premila 2016). They feed on large numbers of small sized and soft bodied prey but harmless to field crops being not herbivorous (Pearce and Zalucki 2006, Chatterjee et al. 2009, Rezac et al. 2010, Hakeem et al. 2018).

Pesticides are highly successful at killing pests, but they also unconsciously reduce the nontarget organisms and natural predators of insect pests including spiders (Amalin et al. 2000, Deng et al. 2006, Cole et al. 2010). Spiders are highly at risk to pesticides that are being used in agricultural fields injudiciously (Pekar 2013). These chemicals affect their longevity, reproduction, defense, development, physiology, mobility, and activities of enzymes (Stark and Banks 2003, Moura et al. 2006, Tahir et al. 2012, Miao et al. 2014, Ndakidemi et al. 2016).

Spiders act as a buffer in agro-ecosystems and keep the pest densities below economic injury level but pesticides reduce their efficiency as biological control agent. Pesticides not only cause direct mortality in spiders but also diminish their efficiency as natural predators (Ataniyazova et al. 2001, Nazarova, 2006, Desneux et al. 2007, Marko et al. 2009, Hanna 2012). Sublethal doses of pesticides weaken the sensory system of spiders and alter their prey choice in the agro-ecosystem (Wrinn et al. 2012, Leccia et al. 2016, Petcharad et al. 2018). After pesticides exposure, these natural predators are unable to differentiate among different types of insect pests. Due to the effect of pesticides, they are unable to capture the most beneficial prey or they feed on toxic prey. Toxic prey consumption diminishes the predator fitness and prey capture potential (Toft 1999, Schmidt et al. 2012).

Acetylcholine esterase (AChE) is a key enzyme that catalyzes the acetylcholine (Oehmichen and Besserer 1982, Wang et al. 2004). Carboxylesterases (CarE) present in insects also detoxifies various chemicals and act as a metabolic activator to various drugs, carcinogens, and ecological toxins. Changes in the activity of AChE and CarE in insects produce metabolic resistance against insecticides (Van Leeuwen and Tirry 2007, Ross et al. 2010, Miao et al. 2016, Jouni et al. 2018, Zeng et al. 2018).

The present study was undertaken to evaluate the effects of λ-cyhalothrin, Bifenthrin (two pyrethroid insecticides), and Glyphosate (herbicide) on the mortality, avoidance behavior, and foraging behavior of *Neoscona theisi* (Walckenaer, 1841), an orb-web spider. This spider species belongs to the family Araneidae and commonly found in agro-ecosystems of Punjab Pakistan (Tahir and Butt 2009). We also studied the effect of insecticides and herbicides exposure on AChE, CarE, and protein contents in spiders.

## Materials and Methods

### Orb-web Spiders Collection and Maintenance

Live adult orb-web spiders were collected from agriculture fields of University of the Punjab Lahore. Spiders were captured by hand picking method. Sampling was conducted from April to August 2018. Adult spiders (only females) from fields were brought to the laboratory in the Department of Zoology, Government College University Lahore. In the laboratory, they were placed individually in separate plastic jars (3 cm wide and 12 cm long) to avoid cannibalism. Mouths of jars were covered with mesh net cloth. Each jar also contained wet soil to maintain humidity at the bottom. Spiders were fed on house flies (*Musca domestica*) in the laboratory. They were acclimatized in the laboratory for 7 d before using them for experiment (Hof et al. 1995). Spiders were identified by Dr. Hafiz Muhammad Tahir, Department of Zoology, GC University Lahore. He is working on spiders from last 15 yr.

### Chemicals

In the present study, we evaluated the toxicity of three commercial pesticides, λ-cyhalothrin, Bifenthrin, and Glyphosate. Recommended field concentrations of λ-cyhalothrin (200 ml/100 liters per acre), Bifenthrin (40 ml/100 liters per acre), and Glyphosate (100 ml/100 liters per acre), respectively, were used in the experiment. The field-recommended doses were used as given in a hand book for agriculture extension agents on the pesticides registered with recommendations for safe handling and use in Pakistan (www.parc.gov.pk).

### Susceptibility Tests

For conducting susceptibility tests against Bifenthrin, λ-cyhalothrin, and Glyphosate, 40 spiders were divided into four groups, i.e., 1) λ-cyhalothrin group, 2) Bifenthrin group, 3) Glyphosate group, and 4) control group. The number of spiders in each group was 10. Whatman filter papers were taken and dipped in the recommended field dose of λ-cyhalothrin (0.5 ml/250 ml of water) Bifenthrin, (0.25 ml/625 ml of water), and Glyphosate (1 ml/100 ml). Filter papers of control group were dipped in distilled water. Separate filter papers were used for each chemical. Filter papers were allowed to air dry for 1 h at room temperature and then placed in petri plates. A single spider was released in each petri plate and allowed to expose to the insecticide, herbicides, or water impregnated filter paper for 1 h. After exposure of 1 h, spider was transferred to the clean jar. No food was provided to spiders during the experiment. The mortality was recorded after every 4 h till 24 h.

### Avoidance Behavior

To investigate the avoidance behavior of spiders, round Whatman’s filter papers were cut into two equal halves. One half of each filter paper was dipped in recommended field concentration of λ-cyhalothrin (0.5 ml/250 ml of water), Bifenthrin (0.25 ml/625 ml of water), or Glyphosate (1 ml/100 ml) while other part of filter paper was dipped in distilled water. Filter papers were air dried for an hour and then both parts were again joined with scotch tape and placed in petri plate. In each petri plate, single spider was released and time (in seconds) spent by each spider on pesticide or distilled water treated part of filter paper was recorded. Spiders were allowed to acclimatize for 15 min before recording the data. For each spider, data were recorded for 30 min (1800 s). Thirty spiders were used in this experiment, 15 for each pesticide. Forty-five spiders were used in this experiment. Spiders were allowed to acclimatize for 15 min before recording the data. The experiment was replicated thrice.

### Foraging Behavior

For this study, following two experiments were conducted.

#### Offering of prey to insecticide-exposed spiders

Adult spiders were divided into experimental and control groups. The number of spiders in each group was 10. Each spider was placed in a container (6 cm wide and 12 cm long). Leaves and twigs were placed in container as anchor points to build a web by spider. To standardized hunger level of spiders, they were first fed with house flies at three different times in the day to the satiation level and then starved for 3 d. Each spider of experimental group was exposed for 30 min to the filter papers treated with sublethal dose of λ-cyhalothrin (one-eighth of field dose), while each spider of control group was exposed to distilled water–treated filter paper. Spiders were exposed to λ-cyhalothrin by releasing them on λ-cyhalothrin impregnated filter paper in closed container. Spiders of both group were offered with same number of prey, *M. domestica* (*n* = 10). Similar experimental set up was used for Bifenthrin and Glyphosate. The sublethal concentrations used for Bifenthrin and Glyphosate were one-sixth and one-third of the recommended field rate, respectively. It was ensured that the size of flies should remain the same for each group. Forty spiders were used in this experiment. The number of flies consumed by spiders of experimental and control group were compared using independent *t*-test.

#### Offering of insecticides exposed prey to spiders

In this, experimental spiders were offered insecticide-exposed prey to the spiders. *Musca domestica* were divided into four groups. Group I, II, and III were exposed to λ-cyhalothrin, Bifenthrin, and Glyphosate, respectively. Group IV was untreated. Similarly, spiders (*n* = 40) were divided into four groups. Each group contained 10 spiders. Each spider of group I was offered prey (*n* = 10) that were treated with sublethal dose of λ-cyhalothrin (one-eighth of field dose) and each spider of Group II and Group III was fed on prey that were treated with Bifenthrin (one-sixth of field dose) and Glyphosate (one-third of field dose), respectively. Group IV was taken as control and fed on untreated prey. The numbers of prey consumed by spiders were recorded till 24 h. The number of flies consumed by spiders of experimental and control groups were compared using independent *t*-test.

### Biochemical Tests

To measure activities of AChE and CarE in insecticide-exposed and unexposed spiders, biochemical tests were performed in the laboratory. Activity of AChE was determined by Ellman et al. (1961) method. CarE activity was measured by Van Asperen (1962) method. Total protein contents were estimated by Bradford method (Bradford 1976).

### Statistical Analysis

Normality of the data was assessed using Shapiro–Wilk test. One-way analysis of variance (ANOVA) test was applied to compare the mortality among different treatments. The number of flies consumed by spiders of experimental and control group were compared using independent *t*-test. Paired *t*-test was used to compare the time spent by spiders on pesticide-treated and control part of filter paper. The predation rate of spiders of control groups and insecticide-treated groups were compared by using ANOVA followed by Tukey’s test for multiple comparisons. Enzyme activity against insecticides/herbicide was compared by using ANOVA. All tests were performed using SPSS (version 22).

## Results

### Susceptibility Tests

**Fig. 1.**
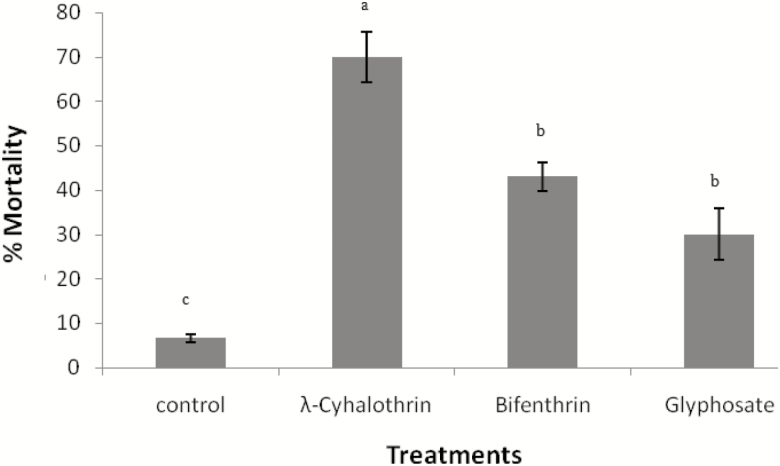
Response of *N. theisi* treated with the recommended field dose of λ-cyhalothrin (0.5 ml/250 ml of water), Bifenthrin (0.25 ml/625 ml of water), and Glyphosate (1 ml/100 ml) in comparison with control group after 24 h post-treatment. Error bars are used to show the standard error.

Highest mortality was recorded against λ-cyhalothrin (70%) followed by Bifenthrin (40%) and Glyphosate (30%). Results of ANOVA showed statistically significant difference among treatments (*F*_3,46_ = 16.00; *P* < 0.05). It is evident from the results of Tukey’s test (Fig. 1) that all pesticide-treated groups showed significantly higher mortality than control.

### Avoidance Behavior

**Fig. 2.**
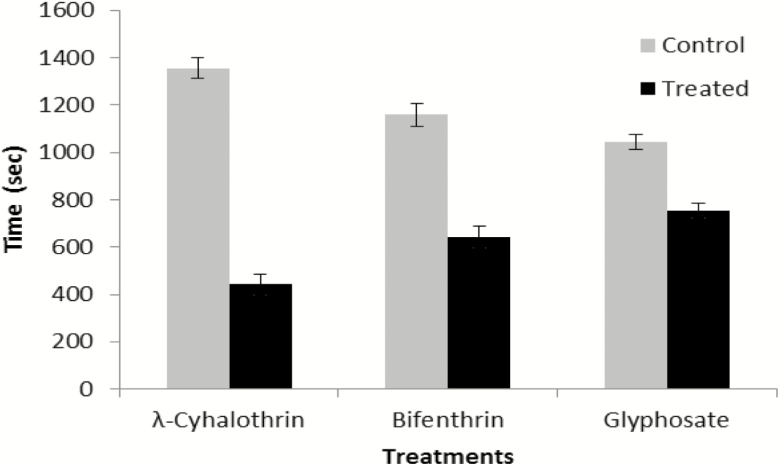
Comparison of total time (seconds) spent by *N. theisi* on λ-cyhalothrin- and water-treated filter paper, Bifenthrin- and water-treated filter paper and Glyphosate- or water-treated filter paper. Error bars are used to show the standard error.

The time (442 ± 44.5 s) spent by *N. theisi* on the λ-cyhalothrin-treated part was less than the time (1,356 ± 44.5 s) spent on untreated part of filter paper. The difference was statistically significant (*t* = −14.5; *P* = 0.001). Time (642 ± 47 s) spent on the Bifenthrin-treated part was less than the time (1,158 ± 48 sec) spent on untreated part of filter paper. Again statistically significant difference was observed (*t* = −7.56; *P* = 0.002). Similarly, *N. theisi* spent less time (754 ± 31.2 s) on Glyphosate-treated part of filter paper than the time (1,044 ± 31.2 s) on untreated part of filter paper (*t* = −8.48; *P* = 0.001, Fig. 2).

### Foraging Behavior

#### Offering of prey to insecticides exposed spiders

**Fig. 3.**
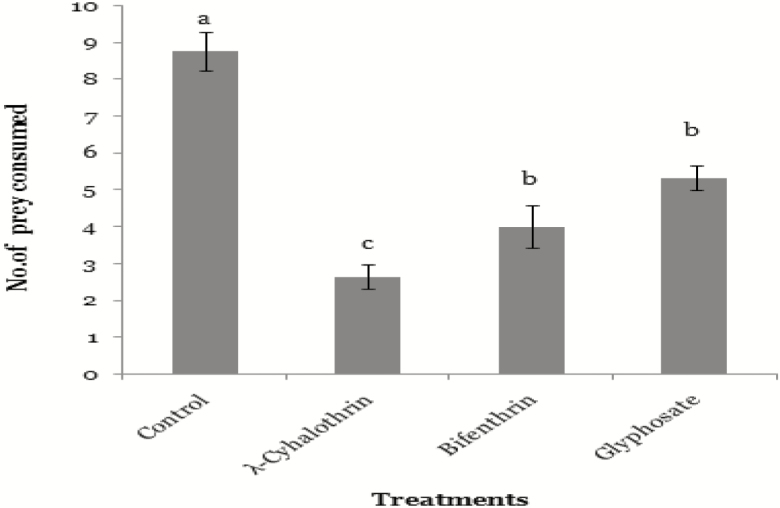
Feeding of *M. domestica* (*n* = 10) by *N. theisi* exposed to λ-cyhalothrin (one-eighth of field dose), Bifenthrin (one-sixth of field dose), and Glyphosate (one-third of field dose) in comparison with control group (24 h). Error bars are used to show the standard error.

Result showed all spiders that were exposed with pesticides consumed significantly less prey than control group of spiders (*F*_3,46_ = 14.34; *P* < 0.05). Prey consumption was lowest in λ-cyhalothrin exposed spiders (2.66 ± 0.33) which was significantly less than the prey consumption of untreated control group (8.33 ± 0.88). The prey consumption of Bifenthrin- and Glyphosate-exposed spiders was 4.00 ± 0.57 and 5.33 ± 0.33, respectively (Fig. 3). Result of Tukey’s test showed that although prey consumption of Bifenthrin- and Glyphostae-treated spiders was significantly as compared control groups but the prey consumption of Bifenthrin and Glyphosate-treated groups differed nonsignificantly (Fig. 3).

#### Offering of insecticides exposed prey to spiders

**Fig. 4.**
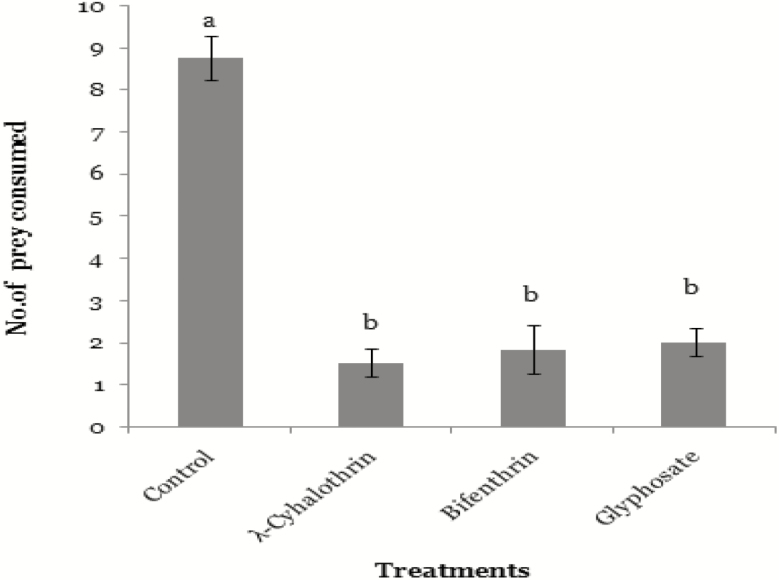
Feeding of *N. theisi* on *M. domestica* (*n* = 10) which were exposed to λ-cyhalothrin (one-eighth of field dose), Bifenthrin (one-sixth of field dose), and Glyphosate (one-third of field dose) in comparison with control group (24 h). Error bars are used to show the standard error.

Overall prey consumption of control and the spiders which were offered pesticide-exposed prey is different (*F*_3,46_ = 11.16; *P* < 0.05). It is further evident from the Fig. 4 that prey consumption of all spiders that were offered pesticide-treated prey differed nonsignificantly. Highest prey consumption was recorded in control group (8.24 ± 0.72), followed by spiders offered with Glyphosate- (1.66 ± 0.12), Bifenthrin- (1.46 ± 0.88), and λ-cyhalothrin (1.33 ± 0.13)-exposed prey, respectively (Fig. 4).

### Estimation of Total Protein (mg/ml)

**Fig. 5.**
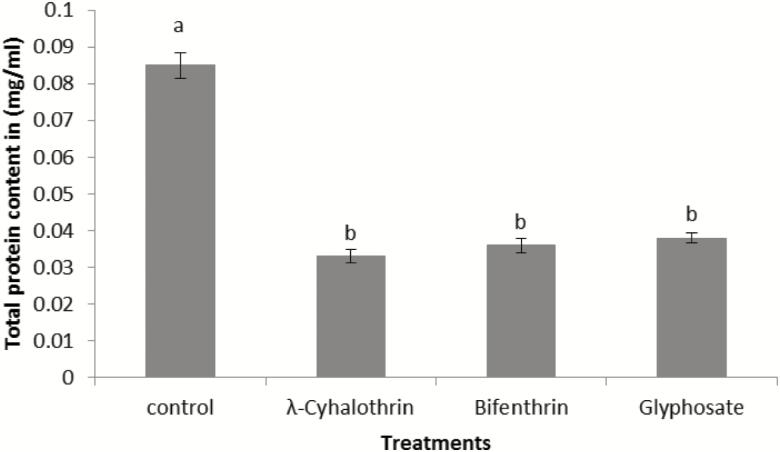
Total protein content in *N. theisi* treated with insecticides/herbicide as compared to control group. Error bars are used to show the standard error.

Total protein contents were decreased in pesticide-treated groups as compared with control group. Significant difference was recorded in the total protein content of treated groups as compared with control (*F*_3,19_ = 102.8; *P* = <0.001). Results of Tukey’s test showed that all treated groups differ nonstatistically but differ from control (Fig. 5).

### Activity of AChE (pmol/min/mg)

**Fig. 6.**
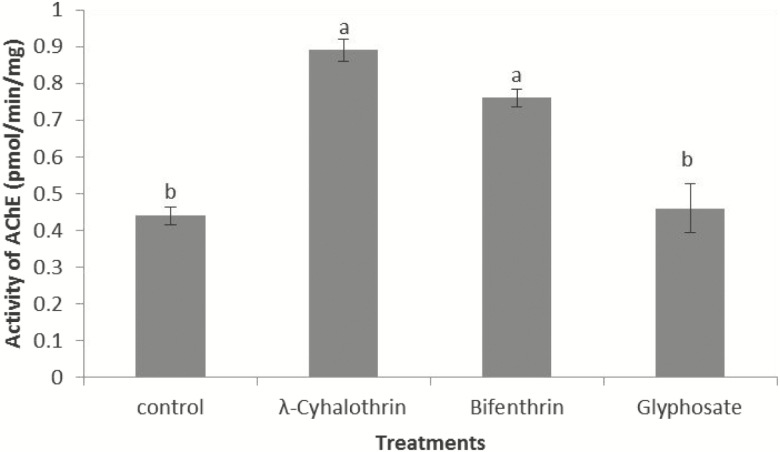
Activity of Acetylcholinesterases in *N. theisi* treated with insecticides/herbicide as compared to control group. Error bars are used to show the standard error.

There was significant difference in the level of AChE in treated group compared with control group (*F*_3,19_ = 3.091; *P* = 0.047). Highest level of AChE was recorded in *N. theisi* treated with λ-cyhalothrin followed by Bifenthrin and Glyphosate, respectively. Lowest level of AChE was recorded in control group. It is evident from Fig. 6 that AChE activity of control- and Glyphosate-treated group differ nonstatistically. Similarly, nonsignificant difference was observed between the activity of AChE in the groups treated with λ-cyhalothrin and Bifenthrin.

### Estimation of activity of CarE (mM/min/mg of protein)

**Fig. 7.**
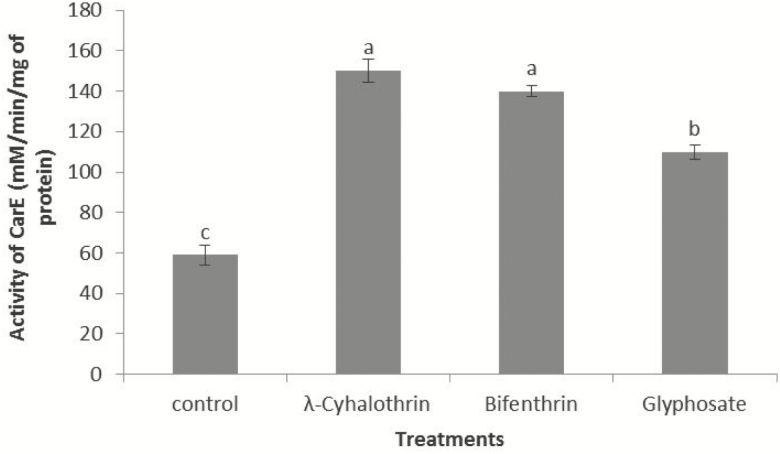
Activity of Carboxylesterases in *N. theisi* treated with insecticides/herbicide as compared to control group. Error bars are used to show the standard error.

There was significant difference in the level of CarE in insecticides treated and control group (*F*_3,19_ = 14.41; *P* = <0.001). Highest level of CarE was recorded in *N. theisi* treated with λ-cyhalothrin followed by Bifenthrin and Glyphosate, respectively. Lowest level of CarE was recorded in the control group. Nonsignificant difference was observed between the activities of CarE in the treated groups. Results of Tukey’s test showed that there is significant difference between control and treated group (Fig. 7).

## Discussion

Pyrethroids are neurotoxic insecticides that are commonly used for the control of different insect pests of field crops to enhance crop yields. They are extremely toxic to insects as they act on the insect nervous system and affect feeding habitat (Yu 2014). Pyrethoids used in agro-ecosystems also affect diversity and abundance of natural enemies (Sherawat et al. 2015). Usually, natural enemies such as spiders are more vulnerable to the effects of insecticides (Li et al. 2014). Application of insecticides results in high mortality of spider in all kinds of agro-ecosystem (Pekar 2012, Bhatti et al. 2013, Rodrigues et al. 2014).

In the current study, effects of two insecticides, i.e., λ-cyhalothrin, Bifenthrin and one herbicide, i.e., Glyphosate on the mortality, avoidance behavior, foraging behavior, and enzymes activity of *N. theisi* was studied in the laboratory. The results of the study showed that selected spider species is susceptible to both insecticides and herbicide. We recorded 70% mortality in *N. theisi* against λ-cyhalothrin. The previous studies have reported that λ-cyhalothrin is highly toxic to the spiders. Khan *et al.* (2017) noted 51% mortality against λ-cyhalothrin in *Plexippus paykulli*, a common jumping spider. Similarly, Dinter (2009) also recorded high mortality in *Erigone atra* after exposing them to λ-cyhalothrin. Tillman and Mulrooney (2000) found that λ-cyhalothrin is toxic to every natural enemy present in cotton.

Bifenthrin was also found toxic to *N. theisi* as it caused 40% mortality at its field rate concentration. Sherwat et al. (2015) reported 65% mortality in wolf spiders of wheat fields, i.e., *Lycosa terrestris* against Bifenthrin. Tahir et al. (2016) observed 80% mortality in *Pardosa sumatrana* at field dose of Bifenthrin. Similarly, Francis and North (2010) also noted high mortality (988.9%) against Bifenthrin among black house spiders. Alzoubi and Cobanoglu (2010) also observed higher mortality in *Phytoseiulus persimilis* after exposing them to Bifenthrin. Wang et al. (2014) reported that Bifenthrin hinders the insect nervous system and causes paralysis, which is usually followed by death.

The observed mortality in *N. theisi* against field rate of glyphosate was 30%. Pereira et al. (2018) recorded 50% mortality in *Cicurina arcuata* against Glyphosate. They further reported that toxicity of Glyphosate is moderate but it causes high irritability. Contrary to Pereira et al. (2018), Benamu et al. (2007) reported that Glyphosate causes no lethal effects on the spider *Alpaida veniliae*. Evans et al. (2010) revealed that Glyphosate exposure affects the behavior and survival of arthropods.

We recorded 80% reduction in prey consumption in *N. theisi* after exposure with sublethal dose of λ-cyhalothrin (one-eighth of field rate) as compared to untreated spiders. Similarly, we noted 60% and 50% reduction in consumption of *N. theisi* which were exposed with sublethal dose of Bifenthrin (one-sixth of field rate) and Glyphosate (one-third of field rate), respectively. Tahir et al. (2015) observed that *Pardosa birmanica* consumed less prey after exposure with λ-cyhalothrin. The changes in prey consumption may be due to several factors, i.e., weak sensory system, altered taste, and altered potency (Michalko and Pekar 2017, Petrakova et al. 2016). Petcharad et al. (2018) reported that insecticides might blur insect’s senses and reduce olfactory capacity; therefore, they do not recognize the prey, which results in less consumption. *Pardosa milvina* females change their predatory behavior when placed on a surface treated with Glyphosate-based herbicide (Wrinn et al. 2012, Sitvarin and Rypstra 2014, Behrend and Rypstra 2018). The interference of pesticides with feeding behavior of exposed insect may include different mechanisms (Desneux et al. 2007).

AChE, CarE, and protein contents were measured in spiders that survive after 24-h exposure of insecticides/herbicide. It was observed that λ-cyhalothrin-, Bifenthrin-, and Glyphosate-treated spiders have increased level of enzymes as compared to untreated control group. Higher levels of these enzymes in the survivors suggest their possible involvement in the metabolic resistance against insecticides/herbicide. Rodrigues et al. (2014) recorded high resistance response in *Eriopis connexa*, which detoxifies λ-cyhalothrin by enzymatic activity. Similarly, Miao et al. (2016) also observed that the AChE activity of *Megacopta cribraria* was significantly increased by LC40 Imidacloprid. Increased esterase level has been recorded in different types of pyrethroids-resistant insect orders, e.g., Hemiptera, Hymenoptera, Diptera, and Lepidoptera (Li et al. 2007, Bass and Field 2011). Recep et al. (2005) also reported that esterase activity of *Tetranychus urticae* was increased against Bifenthrin. Van Leeuwen et al. (2007) observed that λ-cyhalothrin exposure increased the level of CarE activity in *Tetranychus urticae.*

Low level of protein contents were observed in the treated groups of spiders than control group. The results showed that decreased protein contents in treated spiders due to insecticidal stress. Hussain et al. (2009) reported that decreased protein content implies the mobilization of amino acids in insecticide stress to meet energy demands. Similarly, Kliot et al. (2014) reported that metabolic resistance often involves an energy cost in insects and found decreased protein in insects due to energy demand that involved during the activation of detoxification mechanisms and other defense mechanisms.

It is concluded from the study that λ-cyhalothrin is more harmful compared with Bifenthrin and Glyposate. It is suggested that the effect of all pesticides used in agro-ecosystem on beneficial insects should be evaluated before using them in fields. Furthermore, only those compounds should be used in the fields that are pest specific and have least effects on the population of natural predators.
